# The impact of microplastics on female reproduction and early life

**DOI:** 10.1590/1984-3143-AR2023-0037

**Published:** 2023-07-24

**Authors:** Jiayi Yang, Jorke Kamstra, Juliette Legler, Hilde Aardema

**Affiliations:** 1 Farm Animal Health, Department of Population Health Sciences, Faculty of Veterinary Medicine, Utrecht University, Utrecht, Netherlands; 2 Institute for Risk Assessment Sciences, Department of Population Health Sciences, Faculty of Veterinary Medicine, Utrecht University, Utrecht, Netherlands

**Keywords:** microplastics, reproduction, genital tract, oocyte, embryo

## Abstract

Plastic pollution in our environment is one of the most important global health concerns right now. Micro- and nanoplastics (MNPs) are taken up by both humans and animals, mainly via food and water, and can pass important epithelial barriers. Indications of plastics in the blood circulation have recently been shown in both humans and farm animals, but standardized methods to quantify the exact levels of MNPs to which we are exposed are currently lacking. Potential hazards of MNPs are being investigated very recently, including the impact that MNPs may have on reproduction. However, studies on mammalian reproduction are scarce, but a wealth of data from aquatic species indicates reproductive effects of MNPs. The first studies in rodent models demonstrate that MNPs reach the gonads after oral exposure and may impact offspring after maternal exposure during the gestational period. These effects may arise from the particles themselves or the presence of plastic contaminants that leach from plastics. Plastic contamination has been detected in human placentas, fetal fluid and the meconium of newborns, indicating the presence of plastics from the very first start of life. Currently there is a lack of studies that investigate the impact of MNP exposure during the periconception and embryonic period, whereas this is an extremely sensitive period that needs considerable attention with the growing amount of plastics in our environment.

## Introduction

**Figure 1:**
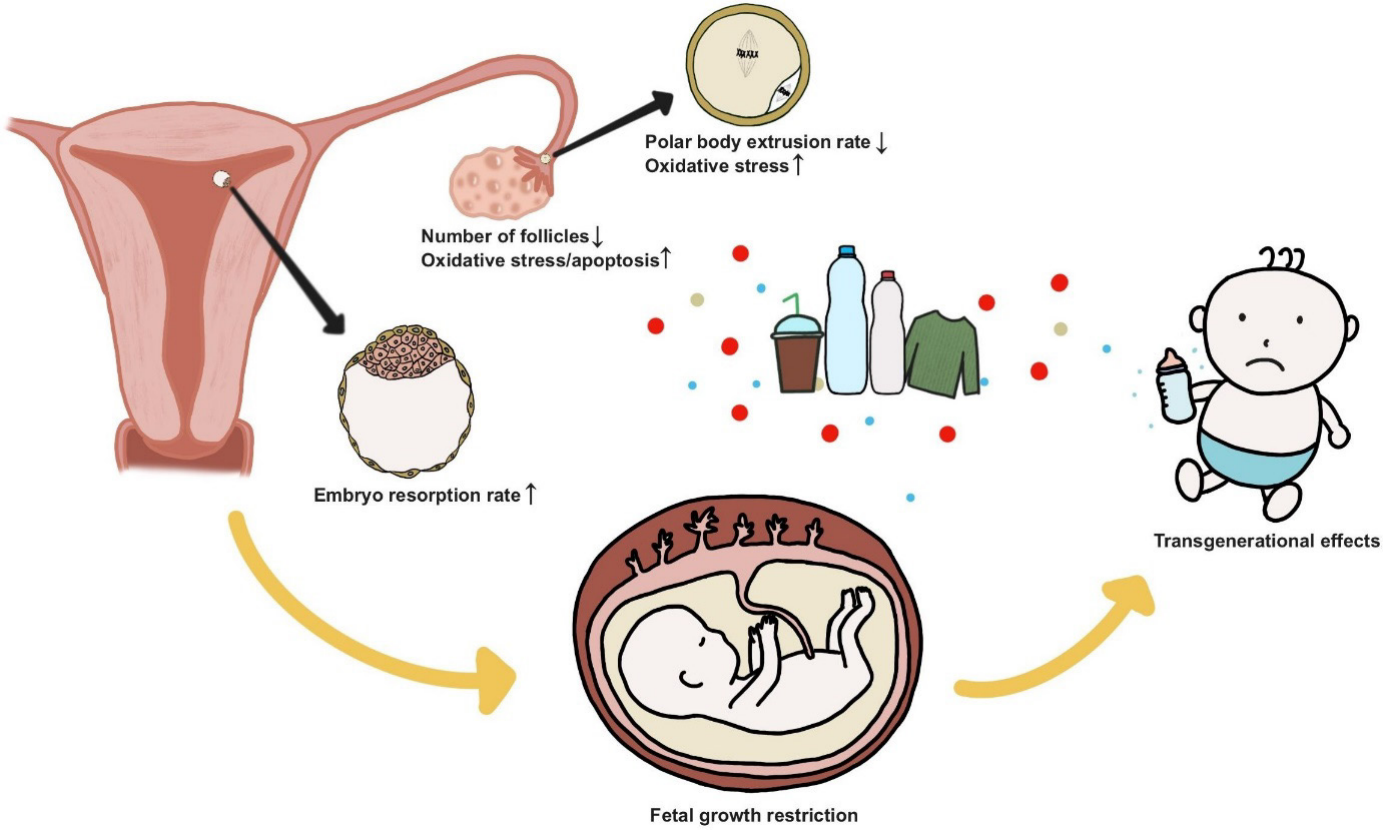
Potential impact of micro and nanoplastics (MNPs) on female reproduction and early life.

Plastic pollution is an increasing global health concern, particularly the ever-increasing amount of tiny plastic particles commonly referred to as micro- and nanoplastics (MNPs). Human activities with a major impact on our ‘global plastic foot print’ are the behavioral and common use of e.g., littering of single-use plastics, packaging and food waste, the release of car tire particles, textile fibers, paint particles, but also inadequate waste management (e.g., open landfills), and environmental conditions (e.g., flooding), including the dispersion of agricultural soils and farmland treated with sewage sludge, and the utilization of agriplastic mulching and fruit protection foams (Huang et al., 2020; Fakour et al., 2021; Yang et al., 2021; Tian et al., 2022). In the environment, plastics remain present for a very long time and disintegrate into smaller pieces, eventually becoming microplastics (MP; < 5 mm) or nanoplastics (NP; < 1 µm), resulting in the current ubiquity of MNPs in global ecosystems. Plastics pollute all environmental compartments, including surface water, sediment, groundwater, soil and the atmosphere (Alimi et al., 2018; Hurley et al., 2018; Ng et al., 2018; Fan et al., 2022; Wang et al., 2021). Recent estimates indicate that we will reach levels of plastic waste of 11,000 Mtons by 2025 (Dong et al., 2023). Unfortunately, once present in the environment, plastics remain in the environment for a long time. Current estimates report that recycling is limited to around 9% of plastic waste, indicating that the remaining 91% of plastic waste enters and remains in the environment (Dong et al., 2023). The widespread presence of MNPs has been demonstrated in many different species and as the environmental levels of MNPs rise, the exposure levels for human, animal, and plant will inevitably increase (Barboza et al., 2018; De-la-Torre, 2020; Azeem et al., 2021). Not surprisingly, plastic pollution has been listed as one of the top 10 environmental problems by the United Nations Environment Programme (UNEP). The UNEP recently issued a statement warning that plastic leaking into farmer’s fields may endanger food security (UNEP, 2022). There are many different types of plastics present in the environment. The most frequently observed polymer types in, for example, freshwater are in decreasing order polyethylene (PE) and polypropylene (PP), polystyrene (PS), polyvinylchloride (PVC) and polyethylene terephthalate (PET) (Koelmans et al., 2019). The presence of MNPs has widely been detected and also appears to be present on human skin and hair, in saliva and sputum, and in stool (Schwabl et al., 2019; Abbasi and Turner, 2021; Huang et al., 2022). Recent data indicates that MPs are even present in blood of humans, and in human urine, breastmilk and placenta, indicating that MPs circulate through the body (Leslie et al., 2022; Pironti et al., 2023; Ragusa et al., 2021, 2022a, 2022b). However, the current lack of standardized methods to analyze the levels of MNPs in different matrices makes it complicated to determine exposure levels of MNP in humans and animals, and to compare different studies (Koelmans et al., 2019; van Mourik et al., 2021). Since, plastics have also been detected in the meconium of new-borns (Zhang et al., 2021), it seems no longer a question whether early life is exposed. To this end, it is important to investigate the potential effects of MNPs on human and animal reproduction, in particular, as some rodent studies show effects of maternal MNP exposure on reproduction (Figure 1). This review aims to give an overview on the current knowledge of the potential effects that MNPs have on female reproduction and early life.

Thus far the rodent model is the most often used model to study the potential impact of maternal MNP exposure in mammals. Studies in rodents show that MNPs reach the genital tract and appear to decrease the number of follicles, while ovarian oxidative stress and apoptosis in granulosa cells are increased. Furthermore, maternal MNP exposure increased the oxidative stress in oocytes and reduced polar body extrusion. In pregnant mice oral MNP exposure resulted in a higher embryo resorption rate and placental and fetal growth restriction, indicating a potential carry-over effect of maternal MNP exposure on offspring. Chemical toxins leaking from MNPs have been demonstrated in human fetal fluid, which stresses the importance to investigate the impact of MNPs on future generations.

### Plastic particles are detected in many species including livestock

Plastic particles can enter the body through ingestion, inhalation, and dermal contact. Ingestion appears to be the main exposure route (Galloway, 2015). MNPs appear to be widely distributed throughout the food chain and have been found in many consumption products like salt, sugar, honey, soft drinks, beer, milk, fruit and drinking water (Pironti et al., 2021). Currently, MNPs and plastic contaminants have been detected in a wide range of different species, with a primary focus on aquatic species, including zooplankton, amphipods, worms, molluscs, fish, sea birds, and whales (Sá et al., 2018). Already back in 2008 it has been demonstrated that the Mussel, Mytilus Edulis, ingested MPs of 3 and 9.6 µm from seawater in an experimental setup (Browne et al., 2008). Mussel ingested MPs ended up in the gut, entered the circulation after 3 days and were present in the mussel until 48 days later (Browne et al., 2008). Lately, the presence of MNPs has also been demonstrated in terrestrial animals, including chickens, sheep, pigs and cattle and appears to originate from their direct (food) environment via plastic waste, via agricultural fields with plastic mulch, food fed to chickens and livestock (Huerta Lwanga et al., 2017; Beriot et al., 2021; Wu et al., 2021), or MNP uptake from water resources (Koelmans et al., 2019). The UN Environment Program (UNEP, 2022) recently issued a statement warning that plastic leaking into farmer's fields may endanger food security. Plastics have also been detected in the blood of livestock in a pilot study, demonstrating that MNPs can enter the body after uptake and are able to pass important epithelial barriers and may potentially reach different organs including the genital tract (van der Veen et al., 2022).

### Potential risks of microplastics for cells

Pristine, commercially available spherical, PS in different sizes is currently the most often used plastic type for *in vivo* and *in vitro* studies that investigate the impact of MNPs on cells. Reported cell responses after exposure to MNPs, are an inflammatory and oxidative stress response, which may potentially result in cell damage and apoptosis, depend on dose, exposure time and the type of plastic to which the cells are exposed.

MNPs that enter the body can harm cells via three potential hazardous routes;

1. Physiological effects attributed to the MNP particle itself;
  *In vivo* experimental studies on MNP toxicity in mammalian and other animal species demonstrate systemic exposure and adverse health effects that are related to physical effects of particles, including immunomodulation and apoptosis, the production of reactive oxygen species and peroxidative damage, impaired neurotransmission, metabolic effects as well as continuous inflammatory activation (Vethaak and Legler, 2021). Responses that are in general also observed after in vitro exposures to MNPs (Silva Brito et al., 2022).
2. Hazardous reactions from the leakage of plastic substances from the MNPs;
  Additives such as plasticizers, antioxidants, flame retardants, pigments etc. incorporated during the manufacture of plastic may be leached into body tissues, resulting in induced changes or bioaccumulation (Issac and Kandasubramanian, 2021). Moreover, many of these additives have been reported to be endocrine disrupting chemicals associated with effects on reproduction via estrogenic or (anti)androgenic properties (Wiesinger et al., 2021).
3. Noxious reactions to pollutants and/or pathogens absorbed by microplastics;
  Metals or chemicals that stick to the surface of the plastic, and depending on the physical and chemical properties of a polymer such as diffusivity, surface area, crystalline, and hydrophobic nature the quantity and type are affected (Rochman et al., 2019; Issac and Kandasubramanian, 2021). Besides this, small plastics may be rapidly colonized by microorganisms including harmful pathogens. Biofilms on MNPs have been reported to be different from the ambient environment, meaning that if ingested, plastics may promote a shift in the structure of microorganisms present in for example the digestive tract (Fackelmann and Sommer, 2019; Santos et al., 2022).

MNPs that enter the blood circulation can be transported to tissues in the body. The active uptake of nanoparticles of cells is by endocytosis, via two main mechanisms either phagocytosis, for particles of > 500 nm, and pinocytosis for smaller particles via a clathrin or caveolin mediated route (Zhao and Stenzel, 2018). Both active and passive transfer are potential options for the uptake of MNPs. Smaller NPs can enter cells via passive transfer when they are small enough to pass the pores of membranes (Lai et al., 2022). MNPs taken up by cells accumulate in the cytoplasm, and phagosomes containing particles may fuse with endosomes and end up in lysosomes (Galloway, 2015). Even particles with a size of around 50 µm appear to be able to enter tissues, which was reported after a 30-day oral MP exposure in mice and subsequent MP uptake in the gut (Deng et al., 2020). MPs with a size between 4-20 µm show the highest level of accumulation in the body, according to a recent review of Dong et al. (2023), possibly due to more potential uptake routes for cells, as both phagocytosis and (macro) pinocytosis are options for uptake. However, another reason for the seemingly lower abundance of smaller NPs in tissues could be the limitation of proper detection methods for the smaller MNPs. An *in vivo* study in a zebrafish cell line demonstrated a dynamin dependent endocytosis route for 50 nm NPs and phagocytosis for 1 µm MPs, both the 50 nm and 1 µm MNPs were distributed towards lysosomes after cellular uptake (Sendra et al., 2021).

Apart from the three previously described potential hazards of MNPs, another potential danger is the enhancing impact that MNPs may have during co-exposures with other stressors. In zebrafish, combined exposure to MNPs and an infection with a pathogen significantly decreased the survival rate. Exposure of zebrafish to the pathogen A. hydrophila, alone resulted in a survival rate of 76%, while combined with 50 nm or 1 µm MNPs the survival rate of zebrafish dropped to respectively 29% and 34%, whereas exposure to MNPs alone had no effect on the survival rate of zebrafish larvae (Sendra et al., 2021). Likewise, the survival rate of immunodeficient zebrafish larvae significantly decreased from 92% towards 6% and 43% in the presence of respectively 50 nm and 1 µm MNPs (Sendra et al., 2021). Furthermore, in a study of He et al. in zebrafish, it was shown that the presence of NPs, and not MPs in this study, enhanced the impact of the toxic compound triphenyl phosphate on the male and female gonad and increased the negative effect on spermatogenesis and oogenesis (He et al., 2021). The enhancing impact of MNPs in the presence of other stressors in the examples above, highlights the importance to investigate the potential effect of MNPs also during co-exposures.

### Microplastics enter the testis and ovary

The information that we have today on the potential impact of MNPs on reproduction is predominantly based on aquatic species and soil fauna and, more recently, also studies in rodents (Yuan et al., 2022). Oral ingestion of MNPs appears to be the major route for uptake of MNPs and once ingested in the gut, MNPs can travel through the body (Galloway, 2015). In a study with oviparous zebrafish, 30 days of oral exposure to 70 nm pristine PS resulted in the accumulation of the NPs in intestine, brain and liver, and an abundant accumulation in male gonads (Sarasamma et al., 2020). Furthermore, in Poecilia Reticulata, used as a representative for viviparous fish and better known as Guppy, 30 days of oral exposure of pregnant fish to 23 nm PS-NPs at 50 µg/L resulted in the accumulation of NPs up until the level of the embryo (Malafaia et al., 2022), indicating that the NPs passed important barriers including the genital tract. In zebrafish, 21-day waterborne exposure to 2 mg/L of 46 nm PS-NPs and 5.8 μm PS-MPs had no effect on the ovarian index, which is a major indicator of reproductive activity in fish (He et al., 2021). However, exposure of zebrafish to 46 nm NPs reduced the number of spawned eggs, while 5.8 μm MPs had no effect on spawning number (He et al., 2021).

Studies in rodents reveal that MNPs accumulate in several tissues in the body after oral uptake, including the lung, spleen, liver, kidney, intestine, brain, and uterus (da Silva Brito et al., 2022). The cellular uptake and distribution of nanoparticles significantly depends on the size, shape, stiffness and surface area of the particle (Zhao and Stenzel, 2018), which is presumably also the case for the uptake and distribution of MNPs in cells. In studies with rodents that were orally exposed, from 20-44 days, to varying PS-MNPs sizes of 50 nm, 80 nm, 500 nm, 4 µm, 5 µm and 10 µm at different concentrations (from 0.015 until 30 mg/kg/d), PS-MNP accumulation in testis and ovarian tissue was demonstrated (Liu et al., 2022; Wei et al., 2022; Jin et al., 2021; Hou et al., 2021a, 2021b). This indicates that MNPs are able to pass the blood-testis and blood-follicle barrier. In male mice a 30 and 45-day exposure period to 5 µm PS-MPs (0.1 mg/d) resulted in a reduction in sperm count and increased number of abnormal sperm cells (Wei et al., 2022). These data are in line with a study by Hou et al. where a 35-day exposure period to 5 µm PS-MPs via water, with a daily consumption of 6-7 mL per mouse (at concentrations of 0.1, 1 or 10 mg/L) resulted in a drop of sperm counts, but in this study only the highest concentrations resulted in an abnormal sperm morphology (Hou et al., 2021a. In a study with male mice that were orally exposed to different sizes of PS-MNPs (10 mg/ml) of 500 nm, 4 and 10 µm during 28 days, all groups showed accumulation of PS-MNPs in the testis, a reduced level of testosterone in serum, and a reduction in viable sperm count and an increased number of abnormal sperm cells (Jin et al., 2021). In all three studies exposure to PS-MNPs was linked with an increased inflammatory response in testis tissue, which may be linked to the observed negative impact on sperm cells (Wei et al., 2022; Hou et al., 2021a; Jin et al., 2021). In the study of Wei et al. both male and female mice were orally exposed to 5 µm PS-MPs (0.1 mg/d) for two days, which resulted in a higher accumulation of PS-MPs in ovaries in comparison to testes (Wei et al., 2022).

In rats it has been demonstrated that after oral exposure to 500 nm PS-NPs (concentrations of 0.015, 0.15 or 1.5 mg/kg/d) for 90 days the PS-NPs entered the ovary and reached the cytoplasm of granulosa cells (Hou et al., 2021b). Rats exposed to PS-MNPs showed a reduced number of follicles and AMH levels, and a dose-dependent higher level of malonyldialdehyde and reduced levels of the anti-oxidants glutathione peroxidase, catalase and superoxide dismutase in ovarian tissue, which is indicative for an oxidative stress response (Hou et al., 2021b; An et al., 2021). Furthermore, oral exposure to PS-MNPs at a concentration of 1.5 mg/kg/d increased the levels of the cytokines 1L-18 and IL-1β, suggesting an inflammatory response, and the by MNP induced oxidative stress appeared to activate the Wnt/β-Catenin signalling pathway and apoptosis in granulosa cells (Hou et al., 2021b; An et al., 2021). When granulosa cells were *in vitro* exposed to 500 nm PS-NPs (0.025 mg/ml) for 24h this resulted in an elevated expression of NLRP3 and cleaved caspase-1 and increased level of apoptosis (Hou et al., 2021b). The aforementioned studies suggest potential negative impacts of PS-MNPs on both testis and ovary after oral exposures, and indicate inflammatory and oxidative stress responses in cells after exposure to PS-MNPs. However, we should take into account that the levels of PS-MNPs used in these studies may be far beyond the levels that are present in blood, and that there may be significant cross species differences. Nevertheless, the demonstrated accumulation of MNPs in the ovary and passage of MNPs over the blood-follicle barrier suggests that they are able to reach the oocyte in the ovarian follicle and request for further investigations into the potential effects of MNPs on the oocyte.

### Do microplastics pose a risk for oocytes?

Whether MNPs affect or enter the oocyte presumably depends on the potency of the granulosa and cumulus cells to temper and prevent the exposure of the oocyte to MNPs, and the extracellular matrix that surrounds the oocyte. The potential mechanisms through which MNPs can enter the oocyte are via 1) passage over the zona pellucida or via 2) gap junctional exchange from cumulus cells towards the oocyte. Oocytes of all animal species are surrounded by a thick extracellular matrix, called the vitelline envelop in most animals, which helps to prevent polyspermy and protects the oocyte and early embryo against influences from outside, like the undesired invasion of viruses (Vanroose et al., 2000). In mammalian species this matrix is called the zona pellucida and it consists of glycoproteins that form a network of overlapping meshes (Clarke, 2022). However, apart from the necessary protective shield of the oocyte and embryo against potential harm from the environment, it also prevents the passage of metabolites needed for growth and development. To this end, there is a connection established from the early stages of follicular growth onwards, most presumably from the primary stage when the zona pellucida is formed, which bridges the contact between cumulus cells and the oocyte via transzonal projections (TZPs) formed by cellular extensions that penetrate the zona pellucida (Clarke, 2022). These TZPs appear to be essential for the transfer of small metabolites (< 1 kDa), like pyruvate from cumulus cells as oocytes lack the machinery to break down glucose, RNA transfer and factors like GDF 9 and BMP 15 that support the bilateral communication between the oocyte and adjacent cumulus cells (Gilchrist et al., 2008; Albertini and Barrett, 2003; Macaulay et al., 2014). The average diameter of pores in the zona pellucida of bovine oocytes appears to be 182 nm, suggesting that passage of particles with a size just below 200 nm may be possible (Vanroose et al., 2000). In pig and rat oocytes, these pores appear to be around 50-100 nm (Stringfellow, 2009). The zona pellucida surrounding the oocyte and the transzonal projections most presumably dictate the maximum size of the MNPs that are able to enter the oocyte. Interestingly, the TZPs and thus the exchange and communication between cumulus cells and the oocyte appears to be tightly regulated by hormonal influences, of follicle-stimulating hormone (FSH) and anti-mullerian hormone, which was described in a recent review paper (Buratini et al., 2022). However, apart from the necessity of TZPs between cumulus cells and the oocyte, together forming the cumulus-oocyte-complex (COC), the TZPs also pose a risk for passage of undesired particles like potentially MNPs, which may not be able to pass the zona pellucida without the TZPs. Until the end of final oocyte maturation when cumulus cells are retracted, the TZPs connect cumulus cells and the oocyte.

After mice were orally exposed to large PS-MPs of 5.0-5.9 µm there was a reduction observed in ovarian size in the study of Wei et al. (Wei et al., 2022), but this was not the case in the studies of Liu et al. (2022) where PS-NPs of 790 nm were used, which may relate to a size-dependent responsiveness of organs to MNPs. When mice were exposed to PS-NPs of 790 nm by oral exposure for 35 days (30 mg/kg), this resulted in the accumulation of PS-NPs in uterus and ovary and resulted in a reduction of the number of antral follicles, whereas the ovarian index (ovary/body weight; an indicator of developmental status of gonad) was not affected (Liu et al., 2022). This data is in line with the studies of Wei et al. and Hou et al. where oral gavage during respectively 44 days to 5.0-5.9 µm PS-MPs (0.1 mg per mouse/d) and 90 days exposure to 0.50 µm PS-NPs 0.15 mg/kg/d and 1.5 mg/kg/d resulted in a reduced number of follicles (Wei et al., 2022; Hou et al., 2021b). Furthermore, in the study of Wei et al. a reduced hormone level of estradiol was reported and an increased level of FSH and luteinizing hormone (LH), but whether this is an indirect effect due to the lower number of follicles and lack of negative feedback on the hypothalamic-pituitary system or a direct effect needs further investigation (Wei et al., 2022). In contrast, the study of Liu et al. (Liu et al., 2022) reported a reduction in antral follicles, but no effect on FSH and LH levels after exposure to 790 nm PS-NPs. Additionally, after exposure to PE-MPs of 10-40 µm for 7 times via intratracheal administration (6, 60 µg/administration), or 90 days of oral gavage of 40 µm PE-MPs (3.75, 16, 60 mg/kg/d) there was no specific ovarian histopathological lesion found (Park et al., 2020; Han et al., 2021). This could indicate that not only the size, but also the type of plastic, PS versus PE, may have a distinct impact on the outcome for ovarian exposure. Furthermore, in the above reported studies pristine MNPs were used, whereas current data demonstrate that more environmental relevant weathered MNPs appear to have a more negative impact on cells (van den Berg et al., 2022). The type and size of MNPs, including pristine versus weathered MNPs, and their potential impact on reproduction certainly needs more attention.

MNPs also appear to affect oocyte competence, as oral exposure of mice to 790 nm PS-NPs resulted in a decrease in first polar body extrusion (PBE) rate, which is the parameter for successful oocyte nuclear maturation at the end of maturation (Liu et al., 2022). In line with this, exposure to 50 nm PS-NPs at 100 µg/mL during *in vitro* oocyte maturation in mice also resulted in a decreased PBE rate whereas no effects were observed at lower exposure concentrations (10, 50 µg/mL) (Park et al., 2022). In addition, after exposure to MNPs the oxidative stress and inflammatory response were increased in the ovary and granulosa cells, and the level of apoptosis was increased in granulosa cells (Liu et al., 2022; Wei et al., 2022; Hou et al., 2021b). Furthermore, there was an increase of oxidative stress in the oocyte after MNPs exposure (Liu et al., 2022; Park et al., 2022). However, in the study of Liu et al., there were no differences observed in embryo cleavage and blastocyst rates after maternal exposure to MNPs, which suggests that oocyte developmental competence was not affected (Liu et al., 2022). Recent pilot data in our group demonstrate that bovine COCs take up 50 and 200 nm PS-NPs, which stresses the importance to investigate the effect of MNPs on oocyte developmental competence (Yang et al., 2022).

Co-exposure may increase the potential impact of MNPs. In a study with zebrafish, co-exposure of diethylstilbestrol (DES), a synthetic estrogen, and NPs to zebrafish during 21 days exacerbated the impact of the solitary exposures on the gonads, and affected the hormone levels of estradiol and testosterone, decreased the cumulative egg number and reduced the fertilization and hatchability and significantly increased the number of abnormalities in offspring (Lin et al., 2023). Furthermore, a recent paper in mice demonstrated that metabolic stress significantly increased the impact of MNPs (Okamura et al., 2023). This indicates that apart from studying the direct impact of MNPs on early life, it is of crucial importance to investigate the effect of MNPs in co-exposures and in a more physiological context with other potential (metabolic) stressors.

### Are embryos hampered by MNPs?

In copepods it has been demonstrated that 50 nm, 500 nm and 6 µm pristine PS-MNPs were ingested and affected fertility resulting in dose-dependent mortality in the F0 and F1 generations, and impaired fecundity in response to 500 nm and 6 µm PS-MNPs (Lee et al., 2013).

In female and male zebrafish fed a diet that contained 42 nm PS-NPs, a dose of 1 mg per gram fish, there was no effect observed on reproduction, despite the induced reduction of glutathione reductase in brain, muscle and testis. However, a worrying finding was that the 42 nm PS-NPs were maternally transferred to embryos, during the 7-day parental exposure period that started the day after mating, and were found in the yolk sac, gastrointestinal tract, liver and pancreas of the offspring (Pitt et al., 2018). Another study in zebrafish demonstrated that large PS-MPs of around 160 µm were not able to pass the chorion barrier of zebrafish embryos, and only a few of the smaller 100 nm PS-NPs passed the chorion and were visible in the periovular fluid, with the majority of the lipophilic MNPs adhering to the chorion (Duan et al., 2020). It was only after hatching that 100 nm NPs were taken up by the zebrafish larvae and accumulated in brain, gills, blood, liver and digestive tract. Zebrafish larvae that were exposed to 50nm NPs and 1 µm MPs for 24h at 120 hours post fertilization (hpf), demonstrated accumulation of the MNPs in mainly the gut, but also in the skin and caudal fin, and the smaller 50 nm NPs were also transferred to the eyes of the zebrafish (Sendra et al., 2021). Furthermore, in vivo exposure of zebrafish larvae at 120 hpf resulted in an increased oxidative stress response in mainly the stomach and gut (Sendra et al., 2021). In the viviparous fish Poecilia reticulata the transfer of PS-NPs, after a 30-day exposure period of females to 23 nm PS-NPs at 50 µg/L, towards embryos was demonstrated and resulted in a reduced pregnancy success rate and offspring number (Malafaia et al., 2022). Although the outcomes of these studies with aquatic species at the level of the embryo are interesting, they may considerably differ from the impact that MNPs have on mammalian embryos. Unfortunately, until now there are only a few mammalian studies reported, which are based on rodent models, that investigated the impact of MNPs during gestation.

Female and male mice receiving a daily gavage of 40-48 µm PE-MPs, at a dosage of 3.75, 15, or 60 mg/kg body weight during 90 days, and mated from day 80 till 89, were not hampered in their reproduction, but there was a slightly lower birth weight of the pups after exposure of the parents to the highest dosage of PE-MPs (Park et al., 2020). Another study where pregnant mice (n=9) were intraperitoneally injected with 10 µm PS-MPs during day 5.5 and 7.5 of gestation, with a dosage of in total 250 µg in a 200 μL saline solution, resulted in a significantly higher rate of implanted embryos and a higher percentage of embryo resorption at day 11.5 after MP exposure in comparison to the control (Hu et al., 2021). Resorption of embryos was also observed in another study after intravenous injection of 300 µg 900 nm PS-NPs at days 9.5 and 15 of gestation, but was not observed after intravenous injection of 60 nm PS-NPs in this study (Nie et al., 2021). However, due to the relatively low numbers of mice that were used in both studies it is difficult to draw strong conclusions out of it. There is one early study that investigated the impact of a 24h exposure period to mixed-sized PS-NPs of 40 till 120 nm in 2-cell stage mouse embryos, where no effects were observed on the competence of embryos to develop into a blastocyst after NP exposure (Bosman et al., 2005). At this point there are, as far as we know, no other studies available that investigated the impact of MNPs during the periconception and early embryonic period. Currently, there is a major lack in information on the potential effects of MNPs during early embryonic development in mammals, while this is an extremely sensitive period in early life. Also indicated by a study in chick embryos where exposure to 60 or 900 nm PS-NPs resulted in malformations and congenital abnormalities after early embryonic exposures (Nie et al., 2021).

### Microplastics enter the placenta and reach the fetus

Plastic particles may also reach and pass the blood-placenta barrier, suggested by the presence of plastics associated chemicals, recognized as endocrine disruptors, in human amniotic fluid and the human placenta (Dusza et al., 2022; Dusza et al., 2019; Ragusa et al., 2021, 2022a). MPs of 5-10 µm in size have been detected in different parts of the human placenta, on the fetal side, maternal side, and in the chorioamniotic membranes (Ragusa et al., 2021). In a recent study of Ragusa et al., MPs were located via transmission electron microscope in pericytes and endothelial cells in the chorionic villi, indicating that MPs are able to reach the fetal blood circulation (Ragusa et al., 2022a). Despite the still limited number of studies on the effect of MNPs on the placenta, the findings are concerning. In *in vivo* mice studies, oral exposure to different sizes of PS-MNPs (50 nm, 100 nm, 5 µm) did not affect the placenta weight (Aghaei et al., 2022, 2023; Chen et al., 2023). Whereas in another study with mice intravenous injection of 60 nm or 900nm PS-NPs, of 300 µg at day 9.5 and 15 of gestation, resulted in a decreased placenta weight and reduced weight of the pups at birth, with an earlier exposure to PS-NPs resulting in increased negative effects on placental and birth weight (Nie et al., 2021). Furthermore, the study of Nie et al., demonstrated more cellular damage in both the placental tissue and fetus after exposure to the smaller NPs of 60 nm in comparison to the 900 nm PS-NPs (Nie et al., 2021). The disruption of immune balance in the placenta, demonstrated by the alteration of macrophage polarization, the population of T cells and cytokine secretion on the fetal-maternal interface-decidua was also observed after exposure to 10 µm PS-MPs (Hu et al., 2021). The placenta plays a critical role for the fetus during gestation in the transport of nutrients and oxygen delivery, which is limited by the maternal blood flow, from the mother to the fetus (Ridder et al., 2019). Exposure to 10 µm PS-MPs during the pre-implantation period decreases the diameter of uterine arterioles from the first trimester onwards; and during gestation exposure to 100 nm PS-NPs results in disruption of the coagulation cascade (Hu et al., 2021). The demonstrated changes in uterine arterioles and the effect of MNPs on the coagulation cascade, may both affect the uterine blood supply and may impact fetal growth and survival. Furthermore, exposure to 50 nm PS-NPs or 5 μm PS-MPs during gestation resulted in a shorter umbilical cord length, which has been related to fetal growth restriction (Aghaei et al., 2022). Fetal growth restriction, has indeed been observed after maternal exposures to MNPs (Aghaei et al., 2022; Chen et al., 2023; Ridder et al., 2019). Moreover, it has been suggested that MNPs may alter placental metabolism, which is related to fetal nutrient supply (Chen et al., 2023; Aghaei et al., 2023). The Barker hypothesis, already introduced in 1995, advocates that an abnormal maternal environment during the susceptible periconception and gestational period can predispose offspring for metabolic aberrances and disease in later life future diseases (Barker, 1995). To this end, further investigation on the potential impact of MNPs on early life are vital.

### Transgenerational effects of MNPs on offspring via maternal exposure

Transgenerational effects have been observed in the past with many different stressors, such as stress, radiation and chemical pollution (Babenko et al., 2015; Fitz-James and Cavalli, 2022; Rebuzzini et al., 2022). Even more, these effects are thought to be inherited via epigenetic mechanisms (Fitz-James and Cavalli, 2022), as many of such stressors do not have the capacity to induce genetic damage. Few studies up to now have been conducted to assess genuine transgenerational effects, i.e. an effect not caused by any carry-over effects of MNP exposures, and mostly in aquatic species (Junaid et al., 2023). On the other hand, multigenerational effects, which might involve carry-over effect of maternal PS-MNPs exposure during gestation and lactation to the next generation, have been reported with effects that include aberrant physiologic behaviour in progeny, reproduction and a higher risk for metabolic disorders in studies with rodents (Liu et al., 2022; Jeong et al., 2022; Wei et al., 2022; Luo et al., 2019a, 2019b). Similar to the toxic mode of action of these particles, these multigenerational effects could be due to altered physiology due to particle toxicity, pathogens of chemicals. Although more extensive research to study genuine transgenerational effects of MNPs are warranted, these results indicate that progeny may not be protected against the harm of MNPs.

## Discussion

Despite the world wide health concern of plastic pollution in the environment, there is currently a lack of information on the impact of MNPs on reproduction. Exposure studies in aquatic and rodent models demonstrate that MNPs travel through the body and can reach the gonads. MNPs are able to pass important epithelial barriers and can reach the circulation, also in humans and farm animals. Until now, most studies are performed with pristine PS particles and studies in mice demonstrate that exposure to MNPs can result in an inflammatory and oxidative stress response of the ovary, including the granulosa cells. Depending on the size of the MNP some mice studies show a dose-dependent effect on oocyte nuclear maturation, and smaller NPs appear to be taken up by the oocyte (Figure 1). However, more environmental relevant MNPs, weathered and plastics of distinct sizes and shapes might have a different impact than what has been found in pristine PS particles (Vethaak and Legler, 2021). Today’s information on the impact of MNPs during the embryonic phase is very scarce. However, in a chicken study it was demonstrated that early exposures to PS-NPs dose-dependently resulted in congenital malformations. Furthermore, zebrafish models demonstrate transfer to embryos after maternal MNP exposure, and based on mice models there are indications for a multigenerational effect on offspring after dams were exposed to MNPs. Plastics are indeed taken up by placental cells and are able to pass the blood-placental barrier and reach the fetus, which was demonstrated by the presence of plastic contaminants in human fetal fluid (Dusza et al., 2022; Ragusa et al., 2021, 2022a; Dusza et al., 2019). However a major lack is the current absence of peer reviewed articles with other than rodent models that report on the impact that MNP exposures may have on mammalian reproduction. Our own pilot data demonstrate that 50 and 200 nm PS-NPs are also taken up by the bovine COC, and may indeed impact early life (Yang et al., 2022). Based on the similarities between bovine and human reproduction during oocyte and embryo development, this may also apply to human COCs.

## Conclusion

It is not a question whether we need to be concerned about early life exposures to plastics and the potential effects of plastics exposure on the next generation, as plastics already invade early life processes (Figure 1). However, currently a major burden is the lack of studies that investigate the impact of MNP exposure during the periconception and embryonic period, whereas this is an extremely sensitive period that needs considerable attention. Therefore, with the daily expanding plastic pollution there is an urgent need to better understand the impact of MNPs on reproduction, early life and next generations.
